# Enhancement of Bone Regeneration on Calcium-Phosphate-Coated Magnesium Mesh: Using the Rat Calvarial Model

**DOI:** 10.3389/fbioe.2021.652334

**Published:** 2021-04-29

**Authors:** Shuang Wu, Yong-Seok Jang, Min-Ho Lee

**Affiliations:** Department of Dental Biomaterials, Institute of Oral Bioscience, Institute of Biodegradable Material, School of Dentistry, Jeonbuk National University, Jeonju-si, South Korea

**Keywords:** magnesium mesh, surface modification, calcium phosphate, guided bone regeneration, rat calvarial defect

## Abstract

Metallic biodegradable magnesium (Mg) is a promising material in the biomedical field owing to its excellent biocompatibility, bioabsorbability, and biomechanical characteristics. Calcium phosphates (CaPs) were coated on the surface of pure Mg through a simple alkali-hydrothermal treatment. The surface properties of CaP coatings formed on Mg were identified through wettability, direct cell seeding, and release tests since the surface properties of biomaterials can affect the reaction of the host tissue. The effect of CaP-coated Mg mesh on guided bone regeneration in rat calvaria with the critical-size defect was also evaluated *in vivo* using several comprehensive analyses in comparison with untreated Mg mesh. Following the application of protective CaP coating, the surface energy of Mg improved with higher hydrophilicity and cell affinity. At the same time, the CaP coating endowed Mg with higher Ca affinity and lower degradation. The Mg mesh with CaP coating had higher osteointegration and bone affinity than pristine Mg mesh.

## Introduction

Guided bone regeneration (GBR) is an osteogenesis technique that has been developed from guided tissue regeneration, and it is used for regenerating new bone at sites with insufficient dimensions, heights, and bone volumes. Bone regeneration procedures, such as reconstruction of the bone structure after excision of ameloblastoma and jaw tumors, augmentation of the deficient height of alveolar ridges caused by periodontics, sinus elevation before implantation, and increase in jaw bone size horizontally or vertically before implantation at the site of tooth loss, are required in many patients undergoing oral and maxillofacial or orthopedic surgery (Miura et al., 2012). Using barrier membranes to form a secluded space is the key to this reconstructive procedure because the newly formed bone can be forced to collapse by the upper soft tissue of a large bone defect. Titanium alloys, cobalt–chromium alloys, and stainless steel are widely used as implant materials in traditional surgery. They have excellent mechanical strength, biostability, and durability, but they can cause stress-shielding effects, and additional surgery is often required to remove the implant after healing. Moreover, they can lead to the visualization of artifacts in magnetic resonance imaging and three-dimensional computed tomography technology, which is not conducive to bone structure monitoring and image evaluation (Hargreaves et al., 2011). Compared with the non-absorbable materials, the advantages of absorbable materials are evident. The latter can degrade in a physiological environment, reducing the need for additional removal surgeries and decreasing the incidence of secondary injuries to the wound; therefore, absorbable materials have been widely used in the biomedical field. However, the unpredictable resorption and unstable rigidity of absorbable materials can affect the structural integrity and barrier function of membranes (Rakhmatia et al., 2013). Therefore, a suitable GBR membrane is expected to not only have sufficient rigidity to reserve anatomic space at the site and exhibit bioactivity to boost new bone formation but also be able to degrade after bone regeneration is complete.

Magnesium (Mg) is a promising biomaterial and has attracted considerable research attention due to its excellent biodegradability, biocompatibility, and biomechanical properties (Staiger et al., 2006). Mg is the fourth most abundant metal ion in the human body, half of which is stored in the bone tissue, and the remaining excess ions are excreted through urine (De Baaij et al., 2015). The density of lightweight Mg is approximately 1.74 g/cm^3^, and elastic modulus is 41–45 GPa. Both of these parameters are similar to those of the human bone (1.8–2.1 g/cm^3^, 3–20 GPa) and better than those of commonly used materials, such as titanium alloys (4.4–4.5 g/cm^3^, 110–117 GPa) and stainless steel (7.9–8.1 g/cm^3^, 189–205 GPa) (Walker et al., 2014). Moreover, Mg also plays a regulatory role as a calcium-sensing receptor in the bone and calcium metabolism (Schlingmann, 2007). The history of biodegradable Mg implants began shortly after the discovery of Mg by Sir Humphrey Davy in 1808 (Witte, 2010). In 1878, Huse used Mg wire as a ligature to successfully stop bleeding in three patients and discovered the corrosive characteristics of Mg in the body (Bettman and Zimmerman, 1935). Since then, many studies have attempted to explore the *in vivo* application of Mg as miniscrews and cardiovascular stents (Witte, 2010). However, the rapid degradation of Mg in the physiological environment raises the problem of changing the pH around the implant and releasing gas, which not only reduces the mechanical strength but also increases the metabolic burden on human organs, thereby limiting its clinical applicability. Hence, many studies have focused on improving the corrosion resistance of Mg and its alloys using various methods.

Surface modification and alloying are the main methods used to control degradation and maintain mechanical properties in pure Mg (PM); however, it is difficult to achieve the required degree of corrosion resistance by alloying alone (Tian and Liu, 2015). Therefore, surface modification is used to form a degradable dynamic interface on Mg-based materials, improving biocompatibility, imparting corrosion resistance, and maintaining mechanical strength; ultimately, such materials can be degraded without the release of any toxic by-products. Calcium phosphates (CaPs) have been used as coatings on implant surfaces because of their biocompatibility, bioactivity, and osteoconductive and osteoinductive properties (Jeong et al., 2019). CaP ceramics may also have the ability to induce appropriate host reactions to connect bone and materials via chemical bonding (Surmenev et al., 2014). Additionally, CaPs have chemical composition and properties similar to those of the mineral phase of human bones and teeth (Yang et al., 2005). Recently, CaPs have been successfully coated on AZ31, Mg–Zn, and AZ91D to improve their corrosion performance to different degrees of success (Xu et al., 2012).

The novelty of the current study is that the effect of CaP coating on pure Mg, in the field of surface performance and *in vivo* osteointegration, was studied from the perspective of the material interface, as this is the first step when the implant material interacts with the host. A uniform and dense CaP protective coating was formed on the surface of PM meshes using a simple alkali-hydrothermal treatment. Surface-modified Mg meshes have excellent biocompatibility and improved corrosion resistance compared with untreated Mg meshes. In particular, samples treated for 2 h showed the best biological activity among various groups stratified according to treatment time. Based on the results, the 2-h group samples were selected to further investigate their application potential as biomedical materials using an animal model. The present study aimed to evaluate the performance difference between the Mg mesh subjected to alkali-hydrothermal treatment for 2 h and original Mg mesh, using *in vivo* and *in vitro* tests. The effect of the CaP coating layer on surface wettability, direct cell seeding, and ion release was evaluated. To further confirm and verify the advantages of CaP coating on the Mg surface over the untreated surface, a critical-size rat calvarial defect was used to evaluate the GBR of CaP-coated Mg mesh and the pure Mg mesh. Osteogenesis was assessed via quantitative and qualitative analyses of GBR employing microcomputed tomography (micro-CT) scanning, histomorphometry, and three-dimensional reconstruction.

## Materials and Methods

### Sample Preparation and Surface Modification

Magnesium foils (99.9% high purity) (Goodfellow, England), 100 mm × 100 mm × 0.1 mm in dimensions, were prepared by rolling and used as the substrate. The Mg foil was subjected to laser microprocessing to form a Mg mesh with a diameter of 10 mm and hole diameter of 0.4 mm. According to the American Society for Testing and Materials (ASTM) standard (G1-03), chemical cleaning procedures were performed to remove the surface corrosion products and revitalize the surface. The Mg samples were directly placed into a beaker filled with a solution containing 0.25 mol/L Ca–ethylenediaminetetraacetic acid (EDTA) (C_10_H_12_CaN_2_Na_2_O_8_) and 0.25 mol/L KH_2_PO_4_, and the solution was adjusted to a pH value of 8.9 with NaOH. The alkaline-hydrothermal treatment was conducted at 90°C for 2 h.

The microstructure and morphology of the CaP coating were identified using scanning electron microscopy (SEM; JSM-5900, JEOL, Japan). The cross-section of surface-modified Mg was sputtered with platinum coating, following which the cross-sectional morphological microstructure was observed using field emission scanning electron microscopy (FE-SEM; SU-70, HITACHI, Japan), while the elemental composition of the designated localized area on the cross-section was investigated using energy dispersive spectroscopy with FE-SEM.

### Implant–Body Fluid Interface Analysis

The initial contact between the implant and host is at the interface between the sample and body fluid. The surface property of the implant is an important consideration for its clinical applicability, which can affect the surface wettability, tissue adhesion, and the release of active ingredients of biomaterials. Therefore, the surface difference between PM and coated Mg (CM) was evaluated by the contact angle, direct cell seeding, and ion release tests.

#### Wettability

The hydrophilicity of the sample surface was evaluated by the contact angles of dropping the cell culture medium on prepared samples to determine the wettability of the sample. To prevent oil or pollution from the skin and environment from affecting the sample surfaces, it was necessary to use 70% ethanol to clean the sample placement plane and use forceps to place the samples. Contact angles and images were obtained by the touch drop method using a contact angle analyzer (Phoenix-300 Touch, Surface Electro Optics, South Korea).

#### Cell Adhesion

The mouse osteoblast cell line, MC3T3-E1, obtained from the American Type Culture Collection, was used directly for seeding onto the prepared sample surface to evaluate cell adhesion and the changes in surface morphological. MC3T3-E1 cells were maintained in a culture medium at 37°C in a humidified atmosphere containing 5% CO_2_. Cells were cultured in α-minimum essential medium (Gibco Co., Carlsbad, CA, United States) supplemented with 10% fetal bovine serum (Gibco Co.), 500 U/ml penicillin (Gibco Co.), and 500 mg/ml streptomycin (Gibco Co.).

After sterilizing the prepared samples with ultraviolet (UV) radiation for 30 min per side, the samples were placed in 24-well cell culture plates. Next, MC3T3-E1 cells at a density of 1.5 × 10^4^ cells/ml were carefully seeded onto the sample surfaces (three wells per group). The cultures were incubated at 37°C in a 5% CO_2_ incubator for 3 days. The morphology of the cells adhered to the samples was observed using SEM. The samples were washed twice with phosphate-buffered saline (PBS) and fixed with 2.5% glutaraldehyde (GA) for 2 h at 4°C. After removing GA and washing twice with PBS, the cells were fixed with 1% osmium for 2 h at 4°C. Following dehydration with gradient ethanol (30, 50, 70, 80, 90, and 100%) for 10 min each at 4°C, the samples were ion sputtered and analyzed by SEM.

#### Release Test

Both sides of PM and CM were sterilized by UV radiation for 30 min before performing corrosion tests. The tests were performed by soaking the prepared samples in Earle’s balanced salt solution (EBSS) and incubating at 37°C in 5% CO_2_. EBSS (1×) was prepared following the manufacturer’s instructions of commercial EBSS 10× (E7510, Sigma, United States). Prepared samples were immersed in a solution in which the ratio of media volume to sample surface area is 0.255 ml/mm^2^ [more than the minimum of 0.20 mml/mm^2^ (ASTM International, 2012)]. Mg and Ca concentrations in EBSS, before and after immersion, were quantitatively analyzed using an inductively coupled plasma-optical emission spectrometer (ICP-OES; Agilent 7500a, Agilent Technologies, Wilmington, DE, United States). Samples were collected after by taking out 1-ml aliquots and then replacing them with 1 ml of fresh EBSS. All measurements were performed in triplicate.

### *In vivo* Analyses

Sixteen Mg meshes manufactured were divided into PM and CM mesh groups for *in vivo* studies. Male Sprague–Dawley rats, aged 8 weeks and weighing 250 ± 20 g, were used as experimental subjects. One week before the experiment, the rats were housed in the animal room at a constant temperature, humidity, and standard light–dark schedule to acclimatize them to the environment.

All animal experiments were conducted under ethical clearance, which was approved by the Institutional Animal Care and Use Committee of the Jeonbuk National University, Laboratory Animal Center, Jeonju-si, South Korea (approval number: CBNU 2020-008).

#### Procedures

The surgery was conducted under aseptic conditions. All samples were sent to the hospital disinfection room (Jeonbuk National University Hospital) for ethylene oxide sterilization before performing the *in vivo* tests.

An 8-mm critical size defect was created surgically, as described in previous studies (Wu et al., 2019). After intramuscular injection of 50 mg/kg of Zoletil (Zoletil 50, Virbac Laboratories, France) and 15 mg/kg of xylazine hydrochloride (Rompun, Bayer, South Korea) to induce general anesthesia, the skin at the surgical site was shaved and then disinfected with iodine scrubs. Additional local anesthesia (0.5 ml of 1% lidocaine) was injected on the calvaria to aid the effects of anesthesia and reduce hemorrhaging. A 2-cm incision was created from the lambda to the middle of the nasal bones, and the periosteum was bluntly dissected to expose the calvaria. The critical-size defect was performed using a trephine bur connected to an endodontic motor (X-SMART, Dentsply, Switzerland) under copious saline irrigation. The edges of the 8-mm-diameter defect were checked carefully and washed to remove residual bone debris. Care was taken to avoid damaging the dura or brain under the bone. The defect was completely covered by mesh, and the periosteum was positioned over the mesh and sutured with bioabsorbable silk (5–0 glyconate monofilament, B. Braun, Rubí, Spain), following which the skin was closed using a non-absorbable nylon silk (4/0 blue nylon, Ailee Co., Ltd., Busan, South Korea). To prevent infection, antibiotics (amikacin; Samu Median Co., Ltd., South Korea) were administered subcutaneously for 3 days postoperatively. The rats were sacrificed at 4 and 8 weeks by euthanization with an overdose of thiopental sodium (Choongwae Pharma Corporation, Seoul, South Korea).

#### Micro-CT

The excised blocks, 20 mm × 20 mm in dimensions, were stored in 10% formalin before being dispatched to the Center for University-Wide Research Facilities at Jeonbuk National University. For quantitative and qualitative analyses, micro-CT (Skyscan 1076) was used to examine the dissected specimens at an 18-μm resolution with a 1-mm aluminum filter. The blocks were scanned at 100 kV voltage and 100 μA current with 360° scanning rotation. The three-dimensional images were reconstructed and analyzed using the NRecon reconstruction program and CT-analyzer software (SkyScan, Aartselaar, Belgium). Regions of interest (ROIs) were created by manually drawing in the region of the 8-mm critical defect to distinguish the newly formed bone and Mg mesh. Three-dimensional images were reconstructed using the CTvox program (SkyScan).

#### Histological Analysis

After micro-CT scanning, the blocks obtained in each group were subjected to a series of fixation, staining, and embedding processes for performing histological analysis.

After micro-CT scanning, the blocks were fixed in fresh 10% formalin for 2 days and stained using Villanueva solution (Polysciences, Inc., Eppelheim, Germany). The blocks were then dehydrated with gradient ethanol (80, 90, 95, and 100%) and 100% acetone. To embed the blocks in resin, the blocks were prepermeated with methylmethacrylate (MMA, Yaruki Pure Chemicals Co., Ltd., Kyoto, Japan) under vacuum for 2 h and then infiltrated with the polymerization mixture (PMMA) at 35°C for 3 days followed by 60°C for 1 day. Prepared resin blocks were cut into 0.7-mm-thick slices through the central line of the defect using a low-speed saw (EXAKT 300 CP, EXAKT Technologies Inc., Norderstedt, Germany). Slices were ground to 70-μm thickness for histological analysis using a microgrinding system (EXAKT 400 CS, EXAKT Technologies Inc.). Histomorphometric analysis was conducted using optical microscopy (10× magnification and 30× magnification; EZ4D, Leica, Wetzlar, Germany).

### Statistical Analyses

The data are presented as the mean ± standard deviation (SD). One-way ANOVA with *post hoc* Tukey’s test was performed, and *p* < 0.05 was considered statistically significant.

## Results

**FIGURE 1:**
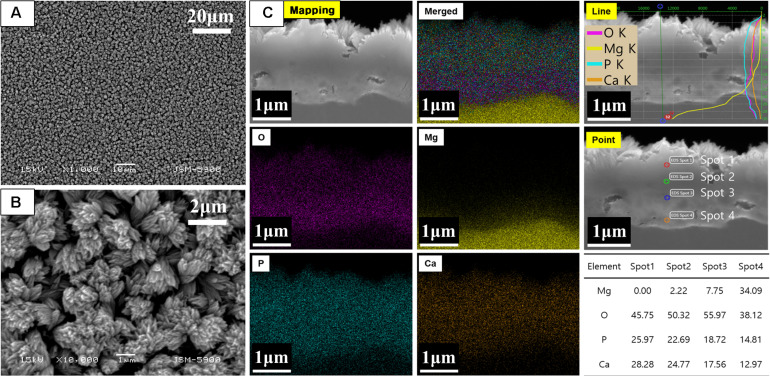
**(A,B)** Surface morphology and **(C)** cross-sectional elemental analysis of CaP-coated Mg after alkali-hydrothermal treatment for 2 h.

Figure 1 shows the results of the surface morphology and cross-sectional elemental distribution of CM. After 2 h of alkali-hydrothermal treatment, the rod-like particles assembled to form a dense and uniform coating layer with a cauliflower-like structure. The thickness of the coating was in the range of 1.5–2.2 μm, and two distinct strata consisting of the dense inner layer and rod-like irregular orientation outer layer were visible. Elemental mapping showed the distribution of Ca, P, O, and Mg in the cross-section. The line profiles of Ca and P showed broad peaks in the outer layer of the coating, while those of Mg and O showed broad peaks in the inner layer. The interface between the Mg substrate and coating was not straight, indicating that a corrosion reaction occurred. The elemental distribution of the point profile showed that Mg mainly existed in the inner layer of the coating, indicating that Mg(OH)_2_ was formed at the interface between the Mg substrate and coating after 2 h of alkali-hydrothermal treatment.

**FIGURE 2:**
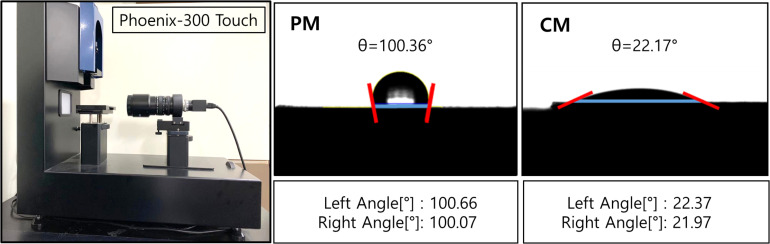
Contact angles of PM and CM were measured using Phoenix-300 Touch, representing the hydrophilicity of the sample surface. PM, pure magnesium; CM, calcium-phosphate-coated magnesium.

The wettability of the sample surface (Figure 2) was evaluated by the touch drop method and presented as optical images and contact angles. The results show that the hydrophilic CaP coating reduced the contact angle of CM to 22.69 ± 2.27°, which significantly improved the wettability of CM compared to that of PM (105.66 ± 7.86°).

**FIGURE 3:**
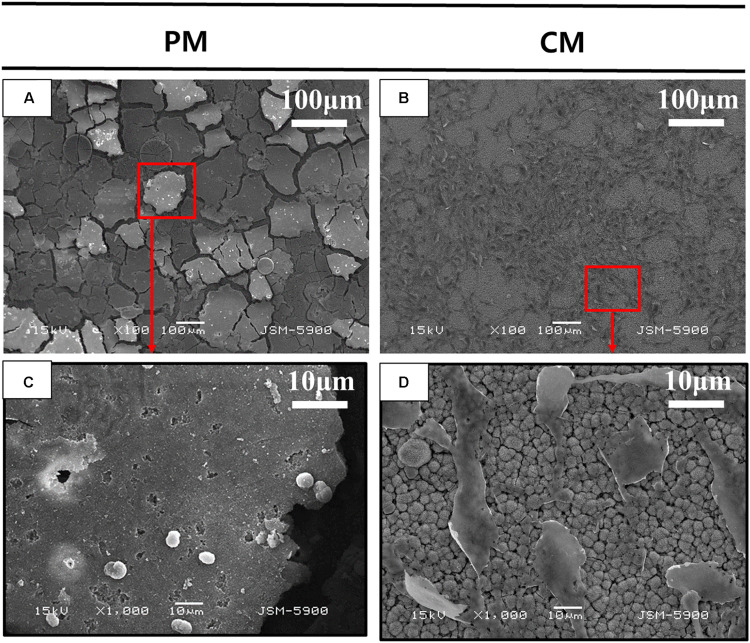
Scanning electron microscopy (SEM) analysis of MC3T3-E1 cells cultured on **(A,C)** pure magnesium (PM) and **(B,D)** calcium-phosphate-coated magnesium (CM) for 3 days. Red color box of A and B enlarged in C and D respectively.

Figure 3 shows the attachment of MC3T3-E1 cells on PM and CM after 3 days of cell culture observed by FE-SEM. Images obtained at 100× magnification showed the attachment of numerous cells and improved cell distribution on the surface of CM compared to that of PM. The morphology of MC3T3-E1 cells cultured on CM showed an elongated shape without filopodia extensions, indicating that the cells were in a state of proliferation. However, in PM, many cells were not found on the surface; in addition, the material appeared to be severely degraded. The few cells detected on PM were white in color and round in shape, indicating cell death and detachment.

**FIGURE 4:**
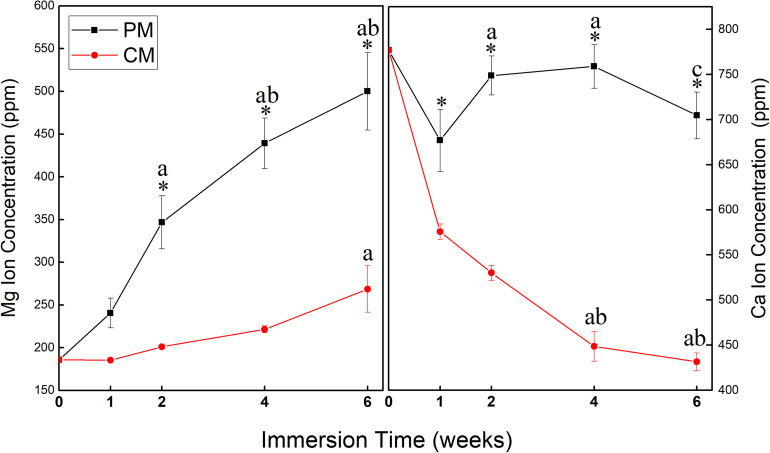
Changes in the concentration of Mg and Ca ions in Earle’s balanced salt solution (EBSS) during the 6-week ion release test of pure magnesium (PM) and calcium-phosphate-coated magnesium (CM) (* indicates significant difference between PM and CM; a indicates significant differences compared to 1 week; b indicates significant differences compared to 2 weeks; c indicates significant differences compared to 4 weeks).

Figure 4 shows changes in the concentration of Mg and Ca ions in EBSS during the 6-week ion release test of PM and CM. ICP-OES test data showed that the release amount and rate of Mg ions from PM were significantly higher than those from CM, indicating that CM had excellent corrosion resistance. At 1 week of immersion in EBSS, the concentration of Ca ions in the PM (677.04 ± 34.46) and CM (575.82 ± 8.50) groups was lower than that in EBSS initially (776.88 ppm). However, the concentration of Ca ions in the PM group began to increase after 1 week, which indicated that deposited Ca ions continued to be released along with the degradation of PM. The concentration of Ca ions in the CM group continued to decrease throughout the test period, which indicated that CaP coating significantly improved the corrosion resistance of Mg and made surface-modified Mg with favorable calcium affinity.

**FIGURE 5:**
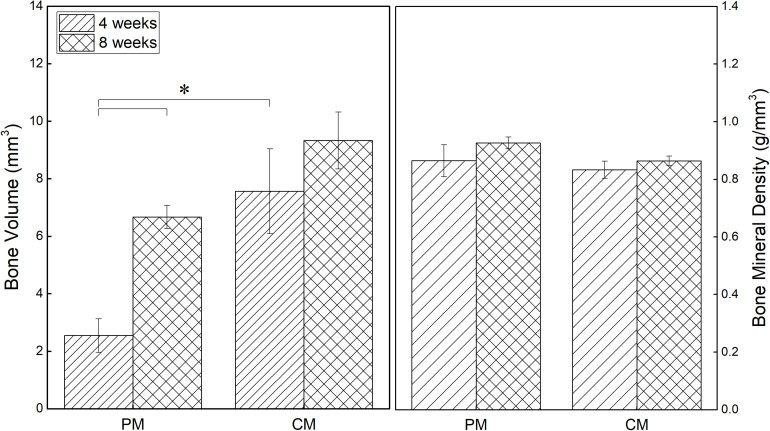
Quantitative analysis results of new bone volume and mineral density in the critical-size defects of rat calvaria obtained from micro-CT data (*p ≤ 0.05).

Quantitative assessment was performed using micro-CT to investigate bone reconstruction within the ROI in rat calvaria. As shown in Figure 5, both the new bone volume and mineral density in the 8-mm critical-size defect of rat calvaria placed with PM and CM increased with time. New bone volume in the CM group was significantly higher than that in the PM group at 4 weeks postsurgery (*p* ≤ 0.05). From 4 to 8 weeks, even if the amount of new bone formation in the CM group increased significantly, there was no significant difference compared with the PM group. In terms of bone mineral density, there was no significant difference between the CM and PM groups.

**FIGURE 6:**
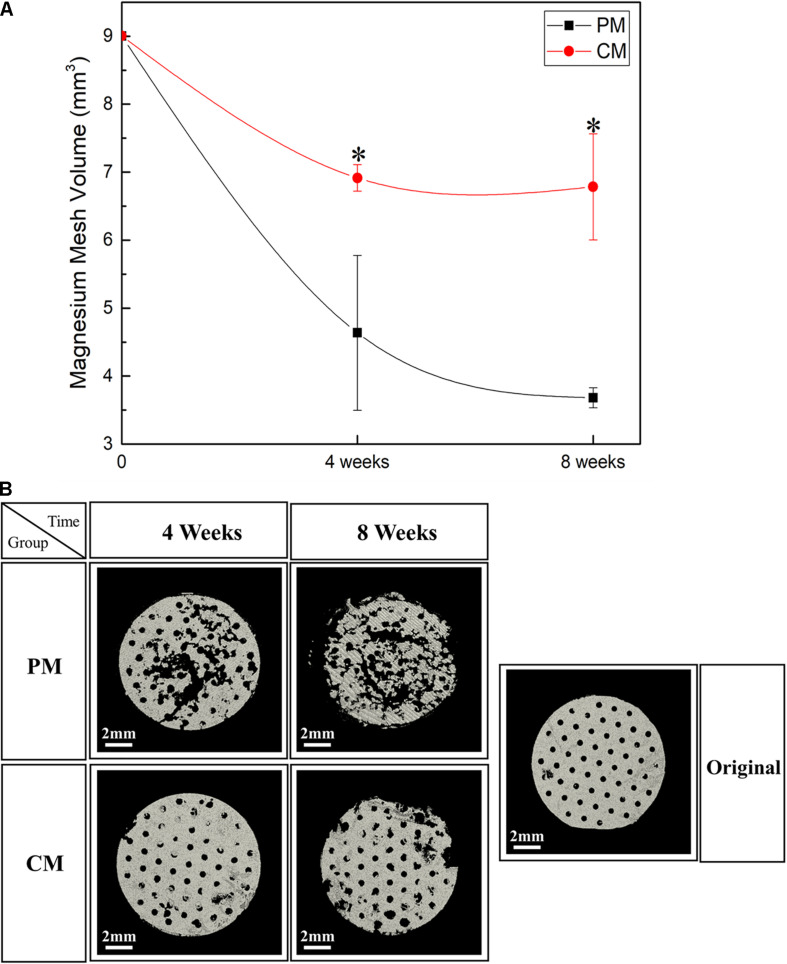
**(A)** Quantitative analysis results and **(B)** three-dimensional reconstruction images of the Mg meshes used in the rat calvarial model for guided bone regeneration obtained using micro-CT [* indicates significant difference between pure magnesium (PM) and calcium-phosphate-coated magnesium (CM)].

To confirm the residual amount of Mg mesh, the ROI was manually drawn to mark the placement of Mg mesh in the rat calvarial model based on micro-CT data. The Mg meshes of all groups showed time-dependent degradation, and the volume of Mg in all groups decreased during each assessed period. As shown in Figure 6, the corrosion speed of PM was fast. The residual Mg volume of PM was 3.74 ± 0.12 mm^3^ at 8 weeks, and the percentage of Mg degradation reached 58.49%. However, the residual Mg volume of CM was 7.02 ± 0.75 mm^3^, and percentage of Mg degradation reached 21.98%. These results were consistent with the three-dimensional reconstructed micro-CT image showing that the PM meshes experienced severe degradation after implantation (Figure 6B).

**FIGURE 7:**
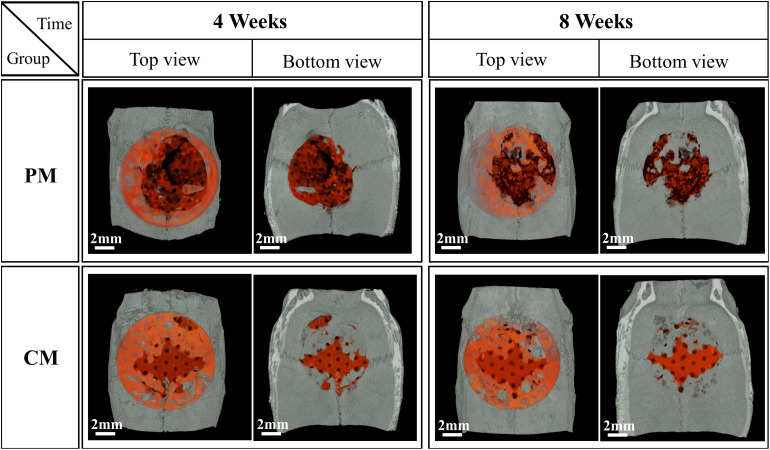
Micro-CT images of the morphology of degraded Mg mesh and newly formed bone in the rat calvaria. The degraded Mg mesh is highlighted in orange.

**FIGURE 8:**
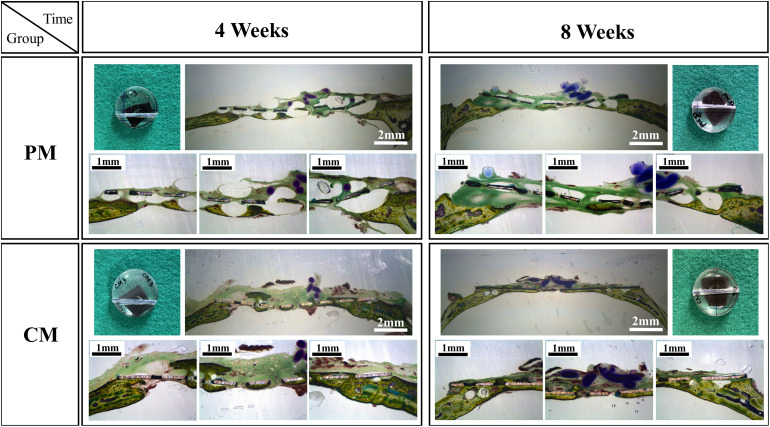
Histological cross-sectional images of bone formation in the pure magnesium (PM) and calcium-phosphate-coated magnesium (CM) groups after 4 and 8 weeks of implantation. Histomorphology is presented as 10× magnification of coronal slices and focal areas of the center and side points (30×).

Figures 7, 8 show bone formation in the rat calvarial defect from the perspective of overall and partial views, respectively. In the three-dimensional reconstruction image of CTvox (see Figure 7), new bone formation can be observed above and beneath the Mg mesh, and both the area of new bone and residual mesh volume in the CM group were higher than those in the PM group. In addition, the partial views in Figure 8 show the relationship between the membrane and new bone in the CM group in which CM led to the generation of a dense and regular new bone layer and also had a direct contact between the mesh and newly formed bones. However, because gas was released when Mg was degraded in the physiological environment, it created a gap between the new bone layer and mesh (Figure 8). In particular, the severe degradation of PM led to a large gap between the new bone and mesh in the PM group.

## Discussion

Previous studies on Mg have mainly focused on the development of Mg alloys to improve surface properties and corrosion protection because alloying is an effective method to improve the mechanical strength of Mg. However, it is difficult to achieve the desired degree of corrosion resistance by alloying alone since thin Mg(OH)_2_ formed spontaneously on the surface can be easily destroyed in the physiological environment including chloride ions, as well as microgalvanic corrosion can occur due to the different potential between Mg matrix and alloy elements (Gusieva et al., 2015). Furthermore, to improve the mechanical strength of Mg alloys, some components such as aluminum (Abd El-Rahman, 2003) are added, which increases the uncertain toxicity of commercial Mg alloys (Song, 2007). Therefore, to improve not only corrosion resistance but also biostability and biosafety, CaP coating was produced on PM, which has been previously confirmed to be effective in controlling early corrosion (Gan et al., 2013) and improving bioactivity (Lorenz et al., 2009).

Recent studies have investigated CaPs as a long-term biocompatibility coating on PM. Zhang et al. (2009) found that biomimetic CaP coating could increase corrosion protection compared with untreated Mg. Gan et al. (2013) showed that bioactive coating with Ca and P on PM was relatively dense and uniform and significantly enhanced corrosion resistance in Hank’s solution. In the study conducted by Lorenz, the protective layer containing CaP was formed by soaking Mg in simulated body fluid for 5 days, leading to favorable initial cell adhesion to samples, but poor protective properties of this layer prevented long-term cell survival (Lorenz et al., 2009). Research on the biocompatibility, biosafety, and biostability of Mg also plays a role in the study of bioabsorbable implants for biomedical applications. It has been shown that amorphous CaP coating on PM mesh significantly retards the biodegradation of PM and improves bone formation in rat calvaria (Wu et al., 2019). A protective CaP coating was formed on PM through a simple alkali-hydrothermal treatment, which not only effectively increased the ratio of Ca and P but also improved corrosion resistance and biocompatibility. In particular, Mg after alkali-hydrothermal treatment for 2 h induced excellent differentiation and proliferation of MC3T3-E1 cells, indicating that Mg has high biocompatibility and biosafety and is suitable for further exploration in bone tissue engineering.

In tissue engineering, the scaffold substrates are mostly dependent on the surface features of materials. The initial interaction immediately occurs between the host tissue and surface of the barrier membrane when the material is placed in the human body and exposed to body fluid, followed by a series of cell–material interactions. Hence, the implant–body fluid interface has a profound impact on the biocompatibility of biomaterials, and the surface properties of materials influence the biological response of the host. Following the application of CaP coating, a series of experiments were performed on the implant–body fluid interface to determine the energy of the surface of Mg in this study. The surface energy generated by the external unsaturated bond is higher than its internal energy. Therefore, when a liquid is placed on a low-energy surface metal, the contact angle is higher than that of a high-energy surface metal (Mekayarajjananonth and Winkler, 1999). When the hydrophobic solid surface of PM with a contact angle of 105.66 ± 7.86° (higher than 90°) changed to a hydrophilic surface of CM with a contact angle of 22.68 ± 2.27°, the CaP coating significantly improved the wettability and surface energy of Mg (Figure 2). Moreover, HAp coating can induce cell attachment, proliferation, and differentiation and increase the activity of osteoblasts (Kim et al., 2014). As shown by the results of the direct cell test (Figure 3), the stable hydrophilic surface of CM has the advantage of inducing cell attachment and cell spreading compared to the hydrophobic surface of PM. This is consistent with previous studies showing that hydrophilic materials with low water contact angles can promote cell adhesion (Ishizaki et al., 2010), spreading (Amornsudthiwat et al., 2013), and proliferation (Watanabe et al., 2012; Kim et al., 2016). Cell adhesion and surface interaction with biomaterials in tissue engineering are mainly attributed to the wettability of materials (Aronov et al., 2006).

During the 6 weeks of the release test, the concentration of Mg ions in the PM group continuously increased to 500.12 ± 45.26, while the concentration of Mg ions in the CM group did not increase (268.56 ± 27.07). This indicates that the release of Mg ions was significantly reduced by the protective CaP coating in the CM group. At 1 week of immersion, Mg ion concentration in the CM group was 185.46 ± 1.87, which was close to the initial Mg ion concentration of 185.81 due to the deposition of Mg ions on the surface of CM owing to their affinity toward electronegative ions such as OH^–^ and PO_4_^3–^. Ca ion concentration in all groups decreased after 1 week of EBSS immersion. In previous studies, CaP coating has displayed bioactivity in simulated body solutions. Ca ions were released into the peri-implant region in the initial stage of implantation, resulting in local supersaturation of Ca ions. Next, the ions were redeposited from the simulated body solution into the implant, which triggered HAp formation (Cazalbou et al., 2005; Paital and Dahotre, 2009). However, the first ion assessment in the current study was carried out after 1 week of immersion, which is markedly beyond the 2 days of immersion assessed in the previous study (Nguyen et al., 2013). This might be the reason why the initial release of Ca ions was not detected in the current study, yet the deposition and concentration reduction in Ca ions were detected (Figure 6). The redeposition layer was a biological apatite layer produced by a biomimetic coating method. Two factors contributed to this layer: (i) CaP coating in CM provided nucleation sites for further deposition of apatite and (ii) the alkaline environment processed into CM provided the necessary functional groups (such as H_2_PO_4_^–^ and OH^–^) for apatite formation (Tanahashi and Matsuda, 1997). As a result (Figure 3D), the structure of CM changed slightly after incubation for 3 days and could induce cell attachment, proliferation, and differentiation to form new bones (Paital and Dahotre, 2009).

After placing meshes for 4 and 8 weeks (Figures 7, 8), a new bone was formed above and beneath the surface-treated meshes and was in close contact. This indicates that the CaP coating has a high affinity for osteoblasts, resulting in osteointegration at the implant–host tissue interface. The degradation of Mg is inevitably accompanied by gas release (Mg + 2H_2_O→Mg^2+^ + 2OH^–^ + H_2_↑), which results in the detachment of newly formed bone or osteoblasts from the mesh. Especially in PM (Figure 8), the large amount of gas produced pushed osteoblasts far away from the mesh, generating a gap between the new bone layer and mesh. After implantation in rat models, the mesh volume of CM and PM decreased after the biodegradation of Mg. By calculating the residual Mg volume, the extent of degradation in CM was lower than that in PM (Figure 6); this is because CM with a protective CaP coating exhibited high corrosion resistance. In addition, excessive Mg ion release during corrosion of the uncoated sample possibly inactivated new bone formation (Serre et al., 1998; Wong et al., 2010), thereby resulting in less new bone formation around the PM compared with the CM. The high osteoblast affinity of coating in CM also contributed to the higher bone volume in CM. Within 4–8 weeks, even if the volume of new bone formed in CM significantly increased, there was no significant difference compared with PM. This may be attributed to the time-dependent degradation of the CaP coating.

## Conclusion

The results of this study demonstrate that the corrosion rate and osteogenesis capability of Mg in the physiological environment can be tailored by using a simple alkali-hydrothermal treatment. CaP coating of Mg favors cell attachment and cell spreading. The formation of a bioactive CaP coating can endow Mg with higher surface energy and osteogenesis capability and lower degradation than PM.

## Data Availability Statement

The original contributions presented in the study are included in the article/supplementary material, further inquiries can be directed to the corresponding authors.

## Ethics Statement

The animal study was reviewed and approved by the Institutional Animal Care and Use Committee of the Jeonbuk National University, Laboratory Animal Center, Jeonju-si, South Korea.

## Author Contributions

SW and Y-SJ conceived and designed the experiments. SW performed the experiments, analyzed the data, and drafted the manuscript. Y-SJ and M-HL revised the manuscript. All authors contributed to the article and approved the submitted version.

## Conflict of Interest

The authors declare that the research was conducted in the absence of any commercial or financial relationships that could be construed as a potential conflict of interest.
